# Exploring the genomic basis of Mpox virus-host transmission and pathogenesis

**DOI:** 10.1128/msphere.00576-24

**Published:** 2024-11-14

**Authors:** Brayden Young, Stephanie N. Seifert, Crystal Lawson, Heather Koehler

**Affiliations:** 1 School of Molecular Biosciences, Washington State University, Pullman, Washington, USA; 2 Center for Reproductive Biology, Washington State University, Pullman, Washington, USA; 3 Paul G. Allen School for Global Heath, Washington State University, Pullman, Washington, USA; University of Michigan, Ann Arbor, Michigan, USA

**Keywords:** *Poxviridae*, Orthopoxviruses, genomic annotation, monkeypox, Mpox, VARV, ECTV, OPG, host range, virulence, transmission, immune evasion, viral evolution

## Abstract

Mpox disease, caused by the monkeypox virus (MPXV), was recently classified as a public health emergency of international concern due to its high lethality and pandemic potential. MPXV is a zoonotic disease that emerged and is primarily spread by small rodents. Historically, it was considered mainly zoonotic and not likely to sustain human-to-human transmission. However, the worldwide outbreak of Clade IIb MPXV from 2020 to 2022 and ongoing Clade I MPXV epidemics in the Democratic Republic of the Congo and surrounding areas are a warning that human-adapted MPXVs will continually arise. Understanding the viral genetic determinants of host range, pathogenesis, and immune evasion is imperative for developing control strategies and predicting the future of Mpox. Here, we delve into the MPXV genome to detail genes involved in host immune evasion strategies for this zoonotic rodent-borne and human-circulating virus. We compare MPXV gene content to related Orthopoxviruses, which have narrow host ranges, to identify potential genes involved in species-specific pathogenesis and host tropism. In addition, we cover the key virulence factor differences that distinguish the MPXV clade lineages. Finally, we dissect how genomic reduction of Orthopoxviruses, through various molecular mechanisms, is contributing to the generation of novel MPXV lineages with increased human adaptation. This review aims to highlight gene content that defines the MPXV species, MPXV clades, and novel MPXV lineages that have culminated in this virus being elevated to a public health emergency of national concern.

## INTRODUCTION

Orthopoxviruses, a genus within *Poxviridae*, infect a wide variety of mammalian species and have been the cause of large-scale human devastation. Orthopoxviruses are enveloped, brick-shaped viruses with a single copy of a double-stranded DNA genome of approximately 200 kb and replicate in the cytoplasm. The middle portion of the genome is highly conserved among other genera within *Poxviridae*, which includes genes crucial for viral translation, replication, and packaging. This genetic arrangement allows for more variability within the left and right terminals of the different virus genomes. These more variable regions contain accessory genes that are not directly involved in essential functions needed to produce functional viral particles. Here, we focus on these accessory genes, as they are critically important in virus-host interaction events that are crucial for understanding Orthopoxvirus pathogenesis.

Up to 50% of the Orthopoxvirus genome is dedicated to regulating the host innate and adaptive immune responses. Within the *Orthopoxvirus* genus, there are 105 common genes involved in host-virus interactions (1). While no single Orthopoxvirus species contains all 105 genes, there are the cowpoxviruses (CPXVs) that contain almost all (1). These genes were initially acquired through waves of horizontal gene transfer events from ancestral hosts and potentially other viruses (1–3). Over the millennia, these genes have undergone selective changes, including mutations, deletions, and duplication events, ultimately giving rise to the major species of Orthopoxviruses (1, 3, 4). Orthopoxvirus species differ in their composition of the left and right accessory gene regions, wherein the function and copy number influence host range and pathogenicity.

While encoding a wealth of virulent factors can offer benefits to viral success, these benefits may be offset by fitness costs. First, a larger genome increases replication time, resulting in more time for the host immune response to recognize and respond to the viral infection. Second, an excess of host-modulating genes can disrupt the immune response excessively, hindering the host’s ability to survive and facilitate successful virion reproduction. Finally, different host species may contain or lack immune pathways that would leave viral genomes with redundant or inessential genes. Consequently, a recurrent pattern among Orthopoxviruses is their active engagement in reductive evolution, minimizing genome size to optimize fitness (1, 5). Eliminating superfluous immune evasion elements, fine-tuning species-specific codon usage, and mutating gene sequences to alter structure-specific binding patterns are all strategies that may narrow the host range while enhancing viral fitness by specializing within a particular host niche.

The host range and pathogenesis of Orthopoxviruses are intricately linked to the presence or absence of virulence genes (6, 7). Among Orthopoxviruses, cowpoxviruses (CPXVs) exhibit the broadest host range, infecting numerous species with varying efficiency consistent with an extensive genomic complement of accessory genes. In contrast, ectromelia virus (ECTV), closely related to CPXVs, specializes in infecting small murine species. Variola virus, with a significantly reduced genome, demonstrates high host specificity, primarily an extremely successful human pathogen. There are too few virulence genes, such as the heavily attenuated modified vaccinia virus Ankara, which experienced excessive ~30-kbp gene loss during repeated *in vitro* passaging, thereby losing the ability to successfully replicate within any mammalian species (8). MPXVs, too, have experienced gene loss, which shaped the species into being a rodent-borne pathogen that has the capacity to effectively infect and be transmitted by humans. The genes that MPXV has lost can, in relation to other Orthopoxviruses, give us clues about the genes unnecessary to establish a niche within primates and Rodentia. Vice versa, Orthopoxviruses that share similar host preferences as MPXV may harbor similar orthopox genes (OPGs) that can provide insights into the important virulence and transmission factors.

Comparative genomics has been widely used to study gene presence and absence related to pathogenesis and host range of Orthopoxviruses (6, 7, 9, 10). Here, we explore the genomic basis of MPXV host specificity and pathogenesis, covering the genes that define immune evasion strategies utilized among the MPXV species and clades. Additionally, we detail how these immune strategies may work to different effects within rodents, mice, and primates to explain the extended host range of MPXV compared to the narrow host range of other Orthopoxviruses. As MPXV has been elevated to a public health emergency of international concern, it is imperative to delineate the important virulence factors and evolutionary mechanisms that are currently fueling its rise (11).

## MPXV SPECIES DEFINING GENES IN RELATION TO OTHER ORTHOPOXVIRUSES

**Fig 1:**
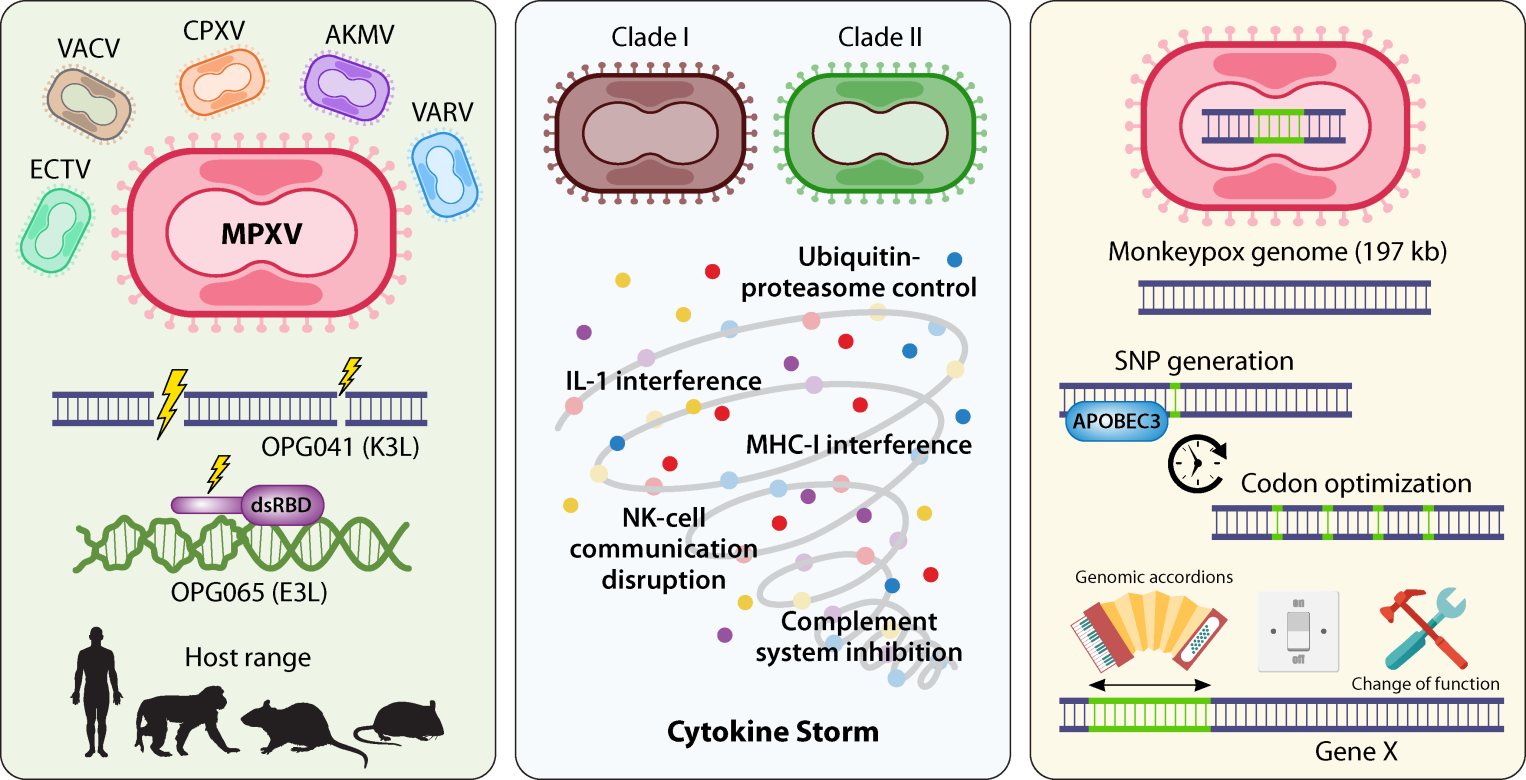
Graphical abstract depicting the three sections of the review: MPXV species-defining traits, MPXV clade differences, and evolutionary forces acting upon MPXV.

Understanding the genes of MPXV is essential for unraveling its pathogenesis, host range, and potential for transmission. The genetic makeup of MPXV, distinct from other Orthopoxviruses, harbors a combination of relinquished and retained genes that play crucial roles in its evolutionary trajectory and interaction with hosts. By examining these genes, we can discern the adaptive evolutionary strategies employed by MPXV to navigate its ecological niche and infect various host species, including humans. In this exploration of relinquished and retained MPXV genes, we delve into the genetic adaptations that shape the virulence, transmission dynamics, and emergence of this significant zoonotic pathogen (Fig. 1).

### Interferon interruption in a species-specific manner dictates host range

For mammalian cells, the activation of protein kinase R (PKR) plays a crucial role in the establishment of an antiviral state. PKR activation has been extensively reviewed regarding its central role in signal transduction through STAT, IRF-1, p53, and NF-κB (12). Importantly, PKR serves as a cytosolic sensor for dsRNA pathogen-associated molecular patterns (PAMPS) that are generated during transcription from dsDNA viruses, a result of both strands of DNA being transcribed simultaneously causing reciprocal RNA binding (12). Importantly, PKR additionally serves to modulate the host translational capability during a viral infection—through the regulation eIF2α (12).

The first genetic difference we detail is a heavily conserved gene among all of Orthopoxviruses and plays a key role in inhibiting the downstream function of PKR. OPG041, also known as Cop-K3L, encodes an eIF2α mimic that interrupts PKR’s ability to modulate the host translational capability during viral infection. Without OPG041/K3L and upon binding to dsRNA, PKR phosphorylates the initiation factor, eIF2α, which competitively inhibits eIF2B resulting in the inhibition of general translation of viral and mammalian mRNAs except for stress-response mRNAs (12). The viral eIF2α mimic blocks the host eIF2α from being phosphorylated, in turn prohibiting viral translation (13). Interestingly, MPXV and ectromelia virus are the only two Orthopoxvirus species that contain a deleterious mutation causing the protein to become non-functional (1, 10). When OPG041 was deleted from vaccinia virus, the virus became attenuated *in vitro* (6). While the IFN response is important for most mammalian species to fight off infection, MPXV and ectromelia virus have adopted alternative strategies for handling down-stream interferon signaling—a potential compensating factor for the loss of function for OPG041.

Vaccinia virus, ectromelia virus, cowpoxvirus, and MPXV contain OPG174, otherwise known as Cop A44L (10). This gene encodes a hydroxysteroid dehydrogenase protein that functions to convert steroid precursors into glucocorticoids, which have immunosuppressive and anti-inflammatory properties, thereby counteracting an IFN-induced anti-viral immune response (10, 14). The deletion of this gene from the Vaccinia virus caused mice to increase IFN production throughout the course of infection (10). The deletion of this gene from the Ectromelia virus or MPXV, in combination with the natural loss of the PKR antagonist, OPG041, may induce a stronger IFN response from the host, thereby attenuating infection. This represents a unique strategy shared by these small-animal-infecting species.

For MPXV, the ability to infect small animal species may depend on the reliance on glucocorticoid production; however, for viruses that infect larger mammalian species, such as primates, the blockage of PKR signaling is a strong selective pressure. Activating the PKR response is a major therapeutic strategy employed by the use of isatin-β-thiosemicarbazone (IBT)—which binds to the Orthopoxvirus RNA polymerase to instigate long mRNA synthesis and subsequent dsRNA PAMPS (15). MPXV is unique in that it has been shown to naturally produce less dsRNA during infection and is IBT-resistant—a result of an unknown mutation within viral RNA polymerases (16). The outcome of this mutation was shown to dampen but not inhibit the PKR response and subsequent IFN production (16). Additionally, OPG034 or VACV Cop-K1L is conserved in ectromelia and MPXV, although fragmented within variola virus. This protein has been shown to reduce the amount of dsRNA during infection, thus weakening PKR activation (17, 18). The combination of genes outlines MPXV’s strategy to overcome PKR activation and together may have allowed MPXV to establish various host niches.

### Hijacking proteasomal degradation to manipulate intracellular signaling cascades

During the extended 24-hour period of Orthopoxvirus replication, intracellular signaling cascades, following PAMP recognition and/or stress responses, are continuously activated and subsequently subverted by interfering viral products (19, 20). A large majority of innate immune signaling cascades coalesce around the ubiquitination of substrates that direct them for activation, translocation, or proteasomal degradation. The host ubiquitin system plays a fundamental role in regulating various cellular processes, including protein degradation, DNA repair, and signal transduction. Ubiquitin, a small protein, is covalently attached to target proteins, marking them for degradation by the proteasome or altering their function, localization, or interactions with other molecules. In the context of viral infections, many viruses have evolved strategies to manipulate the host ubiquitin system to promote their replication and evade immune detection. Here, we detail how Orthopoxviruses utilize ankyrin-like proteins and BTB-kelch proteins dysregulate host proteins, such as NF-κB, through their strategy of interfering host ubiquitin ligases. Additionally, we compare the gene presence and absence of these proteins within MPXV to Orthopoxviruses with restricted host ranges for us to highlight specific ankyrins and kelch-like proteins involved in the pathogenesis of different host species.

First, ankyrin-containing proteins (ANK) and BTB-kelch proteins are major Poxviral gene families associated with interfering E3 ubiquitin ligases (21, 22). E3 ligases are a multi-subunit structure composed of S-phase protein kinase 1 (SKP-1) and Cullin1-3 proteins. Substrates interact with this structure through association with a linker-domain protein, either ANK/F-box, ANK/BC, or BTB-kelch proteins (21). Most poxviral ANK proteins contain an F-box domain, some contain BC domains, and OPG039 (K1L) contains no linker domain (21, 23). Kelch-like proteins also interact with E3 ubiquitin ligases, targeting the cullin 3 ligase, SCF3 (7, 24). Most importantly, ANK and kelch-like proteins have specific targets that are brought to the SCF complexes for ubiquitination and subsequent degradation—although many targets have yet to be found (22).

Overall, poxviruses harbor 14 categories of ANK-containing paralogs (1, 21). Among them, variola and ectromelia viruses exhibit a reduction in half ANK/F-box paralogs, while MPXV has lost five (1). Additionally, BTB-kelch domains are widely dispersed across the Orthopoxvirus species. Variola virus and MPXV have extensive loss of most BTB-kelch proteins (1). Ectromelia, on the other hand, contains three or more BTB-kelch-like paralogs (1, 21). A collection of shared BTB-kelch and ANK proteins is summarized in Table 1.

**TABLE 1:** Selected relinquished and retained OPGs in Mpox virus species and clades^*a*^

OPG	Description	Gene differences in relation to MPXV	VACV-Cop
002	CrmB; TNF-α and other chemokine-binding receptor homolog	MPXV species contains two copies on left and right terminals, VARV only includes one, truncated in ECTV and absent on the right terminal (6, 25).	C22L
003	Ankyrin-containing protein/F-box. Binds to NF-κB subunits	Present in MPXV species. Absent in VARV (7).	C19L
006-14	Bcl-2 domain; PIE domain; BTB domain, no kelch domain; ANK and PRANC domains; C-type lectin domain, putative decoy ligand for NK cell inhibitory receptor; type II transmembrane, TAP inhibitor, inhibits peptide loading on major histocompatibility complex (MHC) I and antigen presentation; BTB and kelch domains; PIE domain, SCP-2; TNF receptor vCD30 homolog, binds CD153, prevents CD30/CD153 interaction; ANK and PRANC domains, binds to cullin 2 and inhibits NF-κB activation	Absent in MPXV and VARV. Largely conserved between ECTV and CPXV.	
015	Ankyrin-containing protein. Interacts with E3 cullin ligase to suppress NF-κB and IRF3 (23)	Truncation in some Clade I MPXV isolates. Absent in VARV (7, 26, 27).	–
016	MHC class I homolog; NKG2D receptor	Inactivation in some Clade I MPXV isolates. Absent in VARV (7, 26, 27).	–
023	ANK/F-box protein and host-range factor; Chinese hamster ovary required factor	*De novo* deletions in MPXV Clade IIb B.1 genomes (28).	–
025	Ankyrin-containing protein	Present in MPXV species, truncated in VARV (7).	C9L
030	Kelch-like protein	Missing in Clade IIa/b MPXV. Present in Clade I. En route to elimination (1, 26).	C5L
032	Complement control protein	Present in MPXV Clade I, VARV, and ECTV. Absent in Clade II MPXVs (6, 29).	C3L
033	Kelch-like protein	Highly diverged from ancestral ortholog (30). MPXV Clade II is further truncated (26). Often lost in connection with OPG32 (1).	C2L
034	TLR-signal inhibitor, Bcl-2 domain	Present in MPXV, VARV, and CPXV. Absent in ECTV.	C1L
038	PIE domain, blocks SD28-mediated T cell activation, secreted	Present in MPXV and VARV species, absent in ECTV species (31).	M2L
039	Ankyrin-containing protein, no F-box	Present in MPXV species and ECTV. Truncation/fragmented in VARV (7, 10).	K1L
041	PKR antagonist, eIF2α homolog	MPXV and ECTV species truncation, predicted nonfunctional (6, 10).	K3L
042	Phospholipase-D like protein	Present in MPXV, CPXV, AKMV, and VPXV. Absent in variola virus.	K4L
043	Monoglyceride lipase	Present in MPXV, CPXV, AKMV, and VPXV. Absent in variola virus.	K5L, K6L
045	PIE domain; blocks apoptosis	Previously absent in Clade II MPXVs, now full-length in both Clades I and II (32). Present in VARV, lengthier in ECTV.	F1L
047	BTB-kelch domain	MPXV Clade I contains full gene, large truncated in VARV (7). MPXV Clade II reported absence (26).	F3L
062	Sequesters RAPTOR and dysregulates mTORC1-mTORC2 signaling	Immunomodulator found within MPXV, VACV, VARV, CPXV, and ECTV (33).	F17
065	Zα/dsDNA-binding protein	MPXV species contains truncation of Za binding domain, non-functional (10).	E3L
067	RNA polymerase subunit	Present in all Orthopoxviruses. Truncated in MPXV Clade II.	E5R
071	DNA polymerase catalytic subunit	More prone to APOBEC3 mutations in Clade IIb MPXV (28).	E9L
105	RNA polymerase subunit	More prone to APOBEC3 mutations in Clade IIb MPXV (28).	J6R
109	RNA polymerase-associated protein	More prone to APOBEC3 mutations in Clade IIb MPXV (28).	H4L
135	Viral membrane-associated, early morphogenesis protein	Low sequence similarity among Orthopoxviruses.	A9L
153	Intracellular mature virion surface tubule protein. Major host antibody target.	STR genomic accordion positioned upstream in Clade IIb genomes (34). More prone to APOBEC3 mutations (28). Frequently lost throughout Orthopoxviruses (1). MPXV clade II more closely related to VARV than Clade I.	A26L
161	EEV envelope glycoprotein	C-terminal truncation in MPXV species.	A33R
174	Hydroxysteroid dehydrogenase; converts steroids into immunosuppressive glucocorticoids	Present in MPXV and ECTV species, absent in VARV species (10).	A44L
183	CrmC; TNF-receptor homolog	Present in MPXV Clade II and ECTV species, absent in MPXV Clade I and VARV species (6).	A53R
188	Poxin-schlafen	More prone to APOBEC3 mutations in Clade IIb MPXV (28). Absent in VACV and VARV (1).	B2R-B3R
192	PIE domain; SCP-3, sequesters cytokines	Present in MPXV and ECTV species, absent in VARV species (10, 25).	B7R
195	PIE, inhibits MHC class I transport	Present in MPXV Clade I, fragmented in Clade II MPXV (7, 29, 35). Present in MPXV Clade I, fragmented in Clade II MPXV and VARV species (7, 29, 35). Absent in VARV and ECTV. Present in CPXV, VPXV, and AKMV.	B9R
198	Serine/threonine kinase homolog	Conserved sequence homology in most Orthopoxviruses except VARV.	B12R
201	IL-1β receptor homolog, secreted, three Ig domains	Present in MPXV Clade I (7). Absent/fragmented in historical MPXV Clade II genomes and VARV (10). New genomes of Clade IIb suggest a lengthier and more efficient protein (32).	B16R
204	IFNα/β binding protein	STR genomic accordion positioned upstream in Clade IIb MPXVs; generated alternative transcription start site (34).	B19R
208	SPI-1; serpin 1	STR genomic accordion positioned start of the reading frame in Clade IIb MPXVs; generated alternative transcription start site (34).	C12
210	PIE domain; surface glycoprotein and T-cell response suppressor	Present in MPXV species and VARV species with high specificity for human T-cells, ECTV ortholog did not inhibit human T-cells (35). Also, a BTB-kelch protein heavily truncated from ancestral OPX (10). Present in MPXV species and VARV species with high specificity for human T-cells, ECTV ortholog did not inhibit human T-cells (35). Also, a BTB-kelch protein heavily truncated from ancestral OPX (10).	–
213	CrmE, TNF receptor homolog	Truncated in MPXV species, further truncated in MPXV Clade IIb (26). Absent in VARV species (36).	–

^*a*^ Orthopoxvirus genes included are present or absent from MPXV in relation to other Orthopoxviruses. OPGs are numbered based on the standard nomenclature (1). Gene names also include the vaccinia virus Copenhagen ortholog gene name. If no VACV name is provided (–), the respective ortholog is not present. VARV, variola virus; VACV, vaccinia virus; AKMV, Akhmeta virus; and VPXV, volepox virus.

Despite a majority of ANK protein substrate targets are unknown, some studies have demonstrated their role in diminishing inflammation through their inhibition of NF-κB. OPG039, an ANK-repeat protein lacking the E3 ligase interaction domain, directly interacts with the p65 subunit of NF-κB, thereby restricting its translocation and subsequently inhibiting the upregulation of inflammatory genes (6, 7, 37). Interestingly, ectromelia and MPXV both contain a K1L ortholog sharing 96% sequence similarity with its counterpart in vaccinia virus. When directly comparing the anti-inflammatory effects of K1L of ectromelia virus compared to vaccinia virus, ectromelia K1L displays a species-specific tropism toward mouse cells (6). This is in contrast to the broader host range exhibited by vaccinia K1L, which is known to function moderately in human and rabbit cells (6, 17). MPXVs ortholog has yet to be studied for tissue tropism preferences defined by the structure of K1L. In total, MPXV carries eight ankyrin-containing proteins, some of which may have host-tropism preferences and therefore may regulate transcription through interfacing with E3 ligase substrates (38). Considering the demonstrated impact on tropism, additional biochemical investigations should be conducted on various poxviral ANK-containing proteins. Defining the substrates targeted by different ANK and BTB-kelch proteins, potentially involving an interplay between host and viral structure, would help delineate host-range determinants and shed light on a potential mechanism allowing for orthopoxviral zoonotic events.

### Cell death subversion and tumor necrosis factor interference

Another large class of virulence factors that participates in host-range differences is the presence or absence of poxvirus immune evasion elements (PIEs). These proteins are important for their structure, rather than their sequence, and are thought to assist with ligand binding (25). PIEs have been rarely lost among Orthopoxviruses and encompass a wide variety of functions, most of which are non-redundant (1, 25). Importantly, the incorporation of different PIEs within species of narrow host range provides insight into the necessary pathways that need to be overcome. For instance, ectromelia and MPXV contain OPG192, which contains a PIE and “secret” domain, which can sequester a host of chemokines (human and mouse CCL28, CCL25, CXCL12b, CXCL13, CXCL14, and mouse CCL23 and CXCL11) (10, 25). This gene is absent/fragmented in variola virus. Another PIE element category that is differentially shared among Orthopoxviruses is the cytokine response modifier, Crm, family. For instance, CrmB and CrmE are both tumor necrosis factor alpha (TNF-α) receptor homologs, OPG002 and OPG213, respectively. TNF-α sequestration from Orthopoxviruses can inhibit cell death cascades and maintain a cellular proliferation and survival state for periods of time to permit replication (24). MPXV and variola virus species both share CrmB, and MPXV and ectromelia virus species share CrmE (5, 6). As there are multiple genes performing the function of TNF-α sequestration, there may be Crm structural differences that exist to encounter TNF-α structures from various host species. Therefore, MPXV contains both OPG002 and OPG213 likely to be utilized to maintain a foothold in reservoirs, small rodents, and human species.

Finally, MPXV is unique from all other Orthopoxviruses in that it has a 37AA N-terminal truncation in OPG065, otherwise known as Cop E3L (39). The E3L protein contains two domains, and the truncation occurs on the N-terminus, which deactivates a Zα domain (39). E3L’s second function, to inhibit PKR sensing of dsRNA, remains unaffected (39). The E3L Zα domain, historically, was shown to bind to Z-nucleic acids, an aberrant left-handed orientation of double-stranded nucleic acid (40). This domain was shown to be an important virulence factor in Vaccinia and Variola viruses, as it functions to subvert host-cell death and evade the type I IFN response (39, 41, 42). During various viral infections, Z-RNA is formed, which can be recognized by host PAMP recognition sensor ZBP1 and initiate an IFN response (43–45). The E3L Zα domain can obfuscate the Z-RNA via direct binding, which hides the PAMP recognition. It is perplexing that both the short and full-length isoforms of the E3L protein are transcribed naturally in Vaccinia, suggesting an additional unknown necessity for the non-functional Zα domain (39).

It is unknown why other Orthopoxviruses have not yet lost or truncated this gene, potentially due to a missing compensatory mechanism that MPXVs possess. One such compensatory mechanism may be that MPXVs are found within OPG003, a viral inducer of RIPK3 degradation. MPXV and ectromelia both contain this gene, whereas vaccinia and variola viruses have truncated or deleted genes, respectively. OPG003 is a kelch-like protein that hijacks the ubiquitin proteasomal system to label target proteins, RIPK3, for degradation (46). The result of RIPK3 degradation is the cessation of the necroptosis cascade and continued cell survival (46). In addition to this product and the fact that MPXV experimentally produces less dsRNA during infection enable it to escape IFN activation and subsequent necroptotic cell death (16).

### Termini of Orthopoxvirus genomes dictate host range and virulence

**Fig 2:**
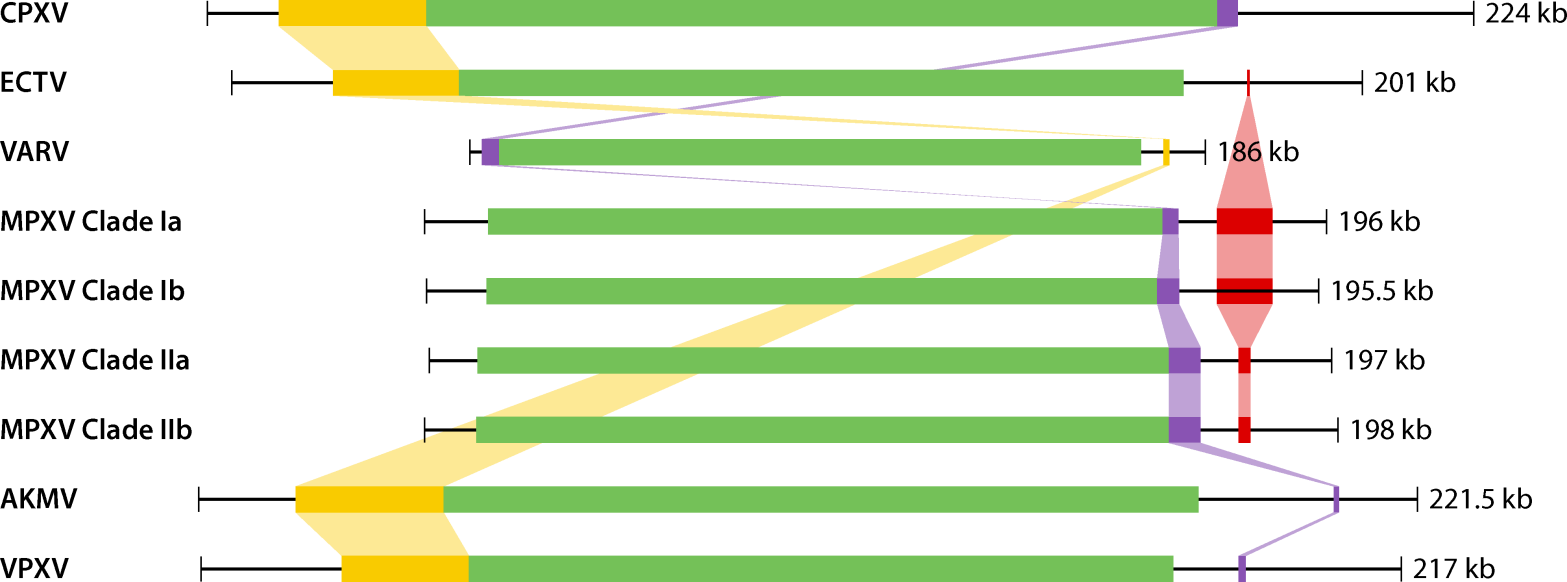
Graphical reconstruction of whole-genome Mauve alignment. Orthopoxvirus genomes were genome aligned via progressive Mauve application within Genious and separately visualized using BioRender. Whole-genome length is depicted using a black line scale. Colored blocks represent regions of the genome sequence that were predicted to be aligned to another genome, presumed homologous. The green block represents the conserved central portion of the Orthopoxvirus genome. Red, purple, and yellow blocks represent regions of shared homology among Orthopoxviruses on the left and right terminals. Reference genomes used are as follows: cowpoxvirus, strain Brighton Red (NC_000363); variola virus, VARV (NC_001611); and ectromelia virus (NC_004105). Clade Ia MPXV (NC_003310) and Clade IIa MPXV (NC_063383) from the Congo (DRC) and West Africa, respectively. Clade Ib MPXV has limited genomic annotations; however, sequences were acquired from recent deep sequencing release, sequenced on 25 December 2023 in South Kivu, DRC (accesion OX044336) (50). Clade IIb MPXV Rivers, 2022 (34). Akhmeta virus AKMV (NC_055230.1) and Volepox virus VPXV (NC_031033.1). Genious files are made available under “Data Availability.”

As stated previously, the middle portion of the Orthopoxvirus genome is heavily conserved. Additionally, the virus-host interaction genes that flank the center are also under positive selection (47). However, the left and right termini of the Orthopoxvirus genome are under constant negative selection and are more readily able to be deleted, duplicated, or inverted (48). Figure 2 is a representation of the Mauve alignment of Orthopoxvirus genomes (raw Mauve alignment files can be found under “Data Availability”). Interestingly, there are regions near the termini of the Orthopoxvirus genomes that are shared and/or lost, corresponding to the host range. The viruses found in rodents and mice, such as Akhmeta virus, volepox virus, ectromelia virus, and cowpoxvirus, all contain a conserved genomic region on the left termini (Fig. 2). The absence of this region from MPXV may be a relic from the separation of Old-World and New-World Orthopoxviruses (49). Therefore, the genes that enable MPXV to be a rodent-borne pathogen are found within the conserved portion of the genome (Fig. 2).

The viruses that are known to cause human disease, cowpoxvirus, variola virus, and MPXV, all contain a conserved region common to the right genome terminus (Fig. 2). The genes of interest within this region contain ankyrin OPG003, IFN-gamma receptor mimic OPG001, apoptosis inhibitor OPG005, and CrmB OPG002 (51). Deletion of this portion within an MPXV-Congo 2003 strain revealed its role in modulating virulence (51). Variola virus also contains this virulence region located at the left termini (Fig. 2). Notably, there is an additional conserved region found among all the MPXVs and not contained in variola (Fig. 2). Genomic surveillance of this region in novel MPXV isolates has revealed considerable volatility (52–54). Whether this region plays a significant role in MPXVs adaptation to humans—or is a byproduct of Orthopoxvirus genomic mutability—remains unanswered.

## MPXV CLADE-SPECIFIC DIFFERENCES

Following the characterization of genes that separated the MPXV genus from other Orthopoxviruses, we expand upon the thesis that gene content infers host range and therefore virulence toward humans. Here, we define gene content differences of Clade I and Clade II MPXVs in comparison to each other. While there are genomic differences within genes involved in replication, transcription, and virion morphogenesis, here we describe the virulence factor genes of importance (Fig. 1). We detail how changes in gene length, through deletion or fragmentation events, have modulated translation and function either to deactivate or increase the efficiency of encoded proteins within alternative host species and transmission contexts.

### Complement control proteins

The Orthopoxvirus gene OPG032 codes for a complement control protein, CCP, and is most notoriously known as SPICE, the small-pox inhibitor for complement enzymes (55). CCPs are secreted proteins that cleave C3b and C4b, which are essential enzymes in the classical and alternative complement pathways, and their absence results in the extended survival of infected cells (10). The Orthopoxvirus CCP is well adapted to mammalian complement, and it likely arose through horizontal gene transfer into an ancestral Orthopoxvirus (6, 56, 57). Most Orthopoxviruses include a CCP that binds to species-specific complement proteins: SPICE for human and baboon complement, vaccinia virus CCP for dog and guinea pig complement, and ectromelia virus CCP for murine complement (10, 58). MPXV, too, contain their respective CCP titled “MOPICE,” the monkeypox inhibitor for complement enzymes.

MOPICE cleaves C3b/C4b into similar products compared to SPICE but performs at the same or marginally better efficiency as vaccinia’s CCP (10, 59). No studies have definitively determined the species specificity of MOPICE. Interestingly, there is a clade distinction in the presence of MOPICE across the MPXV lineages. Clade I MPXV contains the full-length gene, whereas Clade II MPXV have a 10-kbp deletion, which removes the open reading frame and a large portion of MOPICE (29, 60). Even more interesting is the genomic surveillance of recent Clade Ib MPXV lineages within the DRC, which also shows a truncation of MOPICE (61). Removal of MOPICE from Clade I MPXVs reduced disease morbidity and mortality in prairie dog and non-human primate models; however, insertion of the MOPICE gene into Clade IIa MPXV did not increase disease severity (60, 62).

As Clade IIb and Clade Ib infections have a higher transmission rate in humans compared to historical Clade IIa or Ia, MOPICE may be modulating the host-disease susceptibility, thereby weakening transmission potential. This might be an adaptation that favors human-to-human spread by minimizing visible pock lesions and scaring, thus promoting undetected spread. Conversely, the inclusion of the MOPICE could weaken transmission due to modulating systemic and uncontrolled viral spread in the body, thus increasing visible scaring throughout the body. However, as the prairie dog study mentioned prior shows, the inclusion of MOPICE is not a driving factor of virulence within MPXVs; rather, there are additional virulence factors that complement its function, which are present in Clade I rather than in Clade II (60). Therefore, the increased case-fatality rate of Clade Ib, currently spreading in the DRC, is still much higher than the global Clade IIb, driven by virulence factors we review below.

### Adaptive immune response interference

Another strategic difference between the clades is found in their disruption of communication between the innate and adaptive arms of the immune systems. Major histocompatibility complex (MHC) molecules are integral membrane proteins found on the surface of all nucleated cells in the body. They play a crucial role in the immune system by presenting peptide fragments of intracellular proteins to cytotoxic T cells. This process is essential for immune surveillance as it allows the immune system to monitor the intracellular environment for signs of infection or abnormal cell behavior.

Orthopoxviruses contain various strategies to interrupt this immune surveillance, including the disruption of peptide loading, MHC trafficking to the cell surface, and T-cell recognition of MHC (31, 35, 63). MPXV clades differ in their gene sequence of OPG195, an MHC class-1 transport inhibitor (7, 36). MPXV Clade I expresses the full-length gene, whereas Clade II has a fragmented and predicted non-functional protein (51). Interestingly Variola also has this gene fragmented to a non-functional state (36). However, it has been shown that OPG195 is not necessary to disrupt T-cell stimulation, as another gene, OPG210, is able to compensate within MPXV and Variola species (35). OPG210 codes for a glycoprotein that is expressed at the cell surface and contains a PIE domain. It has been shown to suppress T-cell activation through an unknown mechanism (35). As it has been suggested that MPXV’s OPG195 is unimportant for CD4+CD8+ T-cell activation, it may be a gene en route to deletion and/or has alternative unknown functions within Clade I MPXVs (63).

### Intra- and extra-cellular signaling interference

MPXV clades also differ in how they express OPG201, a secreted IL-1β receptor (10). IL-1β is an inflammatory cytokine that, once bound to its receptor, sets off multiple signaling cascades that can lead to cell death, transcription/translation of secreted inflammatory signaling molecules, and promotes an inflammatory immune landscape—all of which act to suppress viral spread. Orthopoxviruses utilize this secreted receptor mimic to inhibit apoptosis, interrupt inflammatory signal transduction, and affect the proliferation of B and T lymphocytes (10, 32). MPXVs differ in the translated length of this protein. MPXV Clade I has contained the full-length OPG201 since the first genomic annotation, whereas historical MPXV Clade II isolates have retained less than a fourth of the total gene length, predicted to be nonfunctional (29).

OPG201 codes for an IL-1β receptor mimic, also containing three immunoglobulin domains that bind to the cell surface to competitively bind to IL-1β. Variola virus has been reported to not contain this gene (7, 36). It has been suggested that Variola suppresses the synthesis of IL-1β far more efficiently and does not require a downstream receptor mimic (7). Additionally, variola virus possesses OPG020, an IL-1 receptor antagonist, whereas each clade of MPXV contains different truncations that result in variable lengths, potentially disrupting this gene’s function (10). Interestingly, new strains of MPXV Clade IIb express OPG201, a lengthier, 180-amino acid protein, which suggests that the IL-1β-binding function has returned to Clade II (32). This new protein product expressed is predicted to bind to IL-1β more efficiently than Clade I’s ortholog (32). It is likely that MPXV clades require this protein due to their inactivated OPG020, suggesting that it is a necessary compensation. Therefore, we should continue surveillance into MPXV’s IL-1 interference genes to identify faults within its strategy as potential therapeutic interventions.

Another interesting distinction between clades of MPXVs is the presence of three kelch-like proteins, orthologs to OPG030, OPG033, and OPG047 within Clade I MPXVs (26). To reiterate, kelch-containing proteins can have a direct effect on E3 Ubiquitin ligase targets, which modulate the host transcriptomic and immune responses. The specific targets that the three proteins target during MPXV Clade I pathogenesis remain unknown; however, their deletion from Clade II MPXVs suggests they are not required for human adaptation (26, 64). Within Clade IIb MPXVs, OPG033 is the most genomically diverged gene from ancestral Orthopoxviruses (30). This suggests an alternative function for the gene or its slow removal. Additionally, this gene has been consistently lost alongside OPG032, the complement control protein (1). The absence/high divergence of both genes from MPXV Clade II indicates a potentially significant interaction between them (1). Understanding why this concurrent loss occurred requires us to know how these two proteins interact within a currently unknown pathway. Further investigation into the roles of kelch-containing proteins would provide insights into their role within different hosts and contexts.

## INTRA-CLADE ADAPTATION AND GENOMIC DIFFERENCES

The spread of MPXV from isolated instances of zoonotic jumps to sustained human-to-human transmission may have been the result of the cessation of Smallpox vaccination (65). Unvaccinated regions became endemic for Mpox, thus establishing the possibility for host coevolution for decades. In this section, we cover two evolutionary mechanisms acting upon MPXV, which “fine-tunes” their gene products to better adapt to human hosts: APOBEC3 mutations and employment of genomic accordions (Fig. 1). Our current knowledge of these mechanisms has been studied within the 2022 global outbreak Clade IIb lineage; however, with the emergence of novel MPXV Clade Ib lineage, these evolutionary signatures are likely to be identified in both strains.

### Single nucleotide polymorphisms altering host tropism

Both the novel Clade Ib and Clade IIb MPXV lineages have extensive APOBEC3 editing signatures across their genome, which have occurred over many years (50, 66, 67). APOBEC3 is a cytosolic mRNA editing enzyme that has been shown to induce C-to-T and G-to-A mutations in DNA viruses including MPXV (68). Due to the recency of these findings, it begs the question as to the overall impact of APOBEC-like editing on the viral evolution of Orthopoxviruses. However, genomic analysis revealed that APOBEC3 mutations are generating more-than-expected numbers of non-synonymous and nonsense mutations across the genome, which follows the trend of reductive evolution within Orthopoxviruses (5, 66). These mutations should be kept under continued surveillance, as frameshifts or nonsense mutations may deactivate genes associated with murine host interaction, which then generates a better human-adapted virus. Additionally, the change of binding pockets through single-nucleotide polymorphisms as well as codon usage change may expand host tropism or restrict the species specificity of virus-host interaction proteins.

There were observed APOBEC3 mutations in MPXV’s Clade IIb B.1 genes: OPG023: ANK/F-box protein and host-range factor; OPG047: BTB-kelch domain contributor of virulence and lesion size; OPG071: DNA polymerase catalytic subunit; OPG105: RNA polymerase subunit; OPG109: RNA polymerase-associated protein; OPG153: an intracellular mature virion surface tubule protein; OPG188: a poxin-schlafen like protein; and OPG210: a surface glycoprotein and T-cell response suppressor (28). While the mutations have not been experimentally determined to be the drivers of increased human-to-human transmission, there are reported codon biases that are preferentially used in human hosts (69). The SNP mutations within ankyrin-containing and BTB-kelch proteins may alter species-specific binding requirements for the stimulation of their target host proteins.

Interestingly, OPG188 was also revealed to be more prone to mutation within MPXV compared to other accessory genes across the genome (28). OPG188 codes for a schlafen-like protein, which are RNA-binding proteins expressed in response to IFN, and plays a role in growth inhibition during an anti-viral state (70). OPG188 also has a conjoined poxin domain that interferes with the STING-dependent interferon pathway via functioning as a cGAMP-specific nuclease (1). Interestingly, the schlafen domain has been truncated, which results in a non-functional variant within variola and vaccinia viruses (1). There is a potential for the higher-than-average mutation rate in OPG188 to create non-synonymous or deleterious mutations that modulate the schlafen-like protein toward inactivation in human-adapted MPXVs, which may increase viral fitness within primate hosts.

### Genome expansion and retraction influence gene length and function

Another interesting evolutionary mechanism, previously experimentally demonstrated within vaccinia virus, has now been found to play a role in the evolution of MPXV Clade IIb strains: the deployment of genomic accordions to modulate gene expression (71, 72). Orthopoxviruses, with their highly mutable genomes, can rapidly expand or contract gene copy numbers during replication cycles through gene duplication or deletion (48, 72–74). In response to selective pressure, the high duplication rate of the Orthopoxvirus genome allows for increases in gene copy number to overcome a host’s pressure (72). There may be toxicity or replication defects associated with a high copy number of specific genes, which may impede the transmissibility of infectious viral particles; thus, the gene copy number can be retracted back to a wild-type state (73). The concept of genomic accordions was first displayed with full genes, such as Cop-K3L (72). Accordion-like poly-A/T repeats have also been identified in vaccinia virus genes (71). Genomic accordions have now also been revealed to occur for short-tandem repeats, which modulate the transcription process of MPXV genes. Short tandem repeats (STRs) are located across the MPXV genome—positioned in intergenic regions or even within coding regions (34, 75). The expansion and retraction in the length of the STRs, much like genomic accordions, can induce frameshift mutations that can turn off, or conversely, turn on genes (71).

In a recent report, Clade IIb MPXVs have been found to utilize genomic accordions of STR regions as well as homopolymer repeats to “fine-tune” their transcription profile (34). There were 21 regions identified that were placed either upstream, downstream, within the reading frame, or right before a promoter region, which displayed variability in length, rather than sequence, among circulating strains (34). There were three major identified accordions that have a functional impact.

The first of which is a STR placed at the start of the reading frame for OPG208, coding for SPI-1, a serine protease that blocks multiple steps of IL-1 processing and cell death cascades (34). This STR produced an alternative start codon, which is transcribed *in vitro*, and incorporated 52 copies of human-associated TAC codons (34). The increased length of the STR more closely resembles the SPI-1 gene in Clade I MPXV (29).

The second is another alternative methionine start codon, built by an STR accordion, upstream of OPG 204, a secreted decoy receptor for type I IFN (34). This alternative start codon is also transcribed *in vitro,* although the functional implications of an extended N-terminal Met-Lys repeat remain unknown.

Finally, there is predicted inactivation of OPG153, a protein that attaches virions to laminin and regulates their egress, which is also a major epitope targeted by the host antibody response (34). This gene contains a poly-D accordion and the insertion of two isoleucines, resulting in a structurally similar OPG153 in Clade IIb, which closely resembles Clade I’s ortholog of OPG153 rather than sister Clade IIa’s ortholog (34). Throughout the history of Orthopoxviruses, this protein has been lost on 18 independent occasions, and its loss has been both shown to attenuate the virus as well as compensate for the loss of a missing transcription factor gene (1). Thus, we should monitor OPG153’s transcription within current Clade I and Clade IIb MPXVs in order to predict its changing epitope or potential for deletion.

### Intra-host variation and a vast genomic landscape promote the potential for novel successful strains

These evolutionary mechanisms provoke the idea of the quasi-species hypothesis. Quasispecies is a concept, often taken from rapidly evolving RNA viruses, which states a viral species is not a monolithic entity but sometimes a diverse population of genomes (76). For Orthopoxviruses, there will be a consensus strain that either initiates the infection or is the most common transmittable product of infection, the “wild type.” However, during replication, Orthopoxviruses are extremely mutable and will generate many defective genomes, as well as potentially generating viruses that have increased copy number or genomic accordions modulating specific genes (48, 73, 77).

Intra-host variation of APOBEC3 editing has been reported previously with Clade IIb (78). The occurrence of localized and systemic disease may be due to changes in the copy number of specific virulence factors of importance within different tissues. Whole genome sequencing at site-specific locations (e.g., multiple locations of skin lesions, nasopharyngeal swabs, or a lymph node biopsy) can aid our understanding of intra-host variants (79). Another instance of this genomic volatility occurred within a Clade I MPXV strain, wherein the genome had a large deletion that caused the truncation of an ankyrin-repeat-containing protein OPG015, and the inactivation of natural killer activating receptor inhibitor, OPG016 (27). This deletion is hypothesized to have increased human-to-human transmission for a short time period (26, 27). Moreover, molecular investigation has revealed that Orthopoxviruses can “steal” and expand genes to gain molecular footholds within related species during zoonotic jumps (74, 80). Such events are microcosms of the larger evolution of Orthopoxviruses. Consistent genomic surveillance in regions where MPXV is endemic, including sharing of raw deep sequencing data, would allow us to better assess the ever-evolving genomic landscape of emerging MPXV variants.

## CONCLUDING REMARKS ON THE FUTURE OF MPXV

Orthopoxviruses are ubiquitous pathogens that infect a wide variety of species and have infected humans since before the domestication of cattle. The genes that Orthopoxviruses contain are specially crafted to interfere with the mammalian host immune responses. These genes disrupt intracellular signaling cascades, interfere with cellular communication and secretion of cytokines, as well as controlling host gene expression. The vast collection of immune evasion genes incorporated within Orthopoxviruses genomes is tailored in different combinations for host specialization. Therefore, for Orthopoxviruses to become better transmittable pathogens, they do not need to just decrease their virulence. Instead, they can first delete unnecessary genes from their genome that are not required in host niche specialization. With the emergence of MPXV—and the eventual arrival of novelly adapted human MPXVs—we can predict the important virulence genes involved in human disease with further investigation of the genes described in this review.

Specifically, the loss of function in OPG041 and OPG065 (K3L and E3L, respectively) is a unique strategy of virulence that distances MPXV from all other Orthopoxviruses. Triggering IFN response in the host, as some have hypothesized, may be an important therapeutic strategy to combat MPXV infection (81). Furthermore, ankyrin-containing proteins play important roles in interfering with the viral-host response, and they influence the host range of Orthopoxviruses. MPXVs that specialize toward human adaptation may, therefore, alter structures of their ankyrin/F-box proteins to target human-specific proteins, while MPXVs that remain primarily zoonotic may induce weakly stimulatory effects on rodent and primate E3 ligases and their substrates. Previous investigations corroborate the fact that viral and host structures are inextricably linked to host range (82, 83). However, currently, there is a dearth of information on species-specific stimulatory effects based on ankyrin/F-box structures. Altogether, the ongoing jump of MPXVs from rodent-borne to becoming effective human pathogens is the result of continuous reductive evolution and refining of immune evasion strategies, which resulted in the establishment of a molecular foothold in human populations.

Transmission within human communities is also modulated by the virulence of MPXV strains. MPXV exists within two clades with major differences in disease presentation and intra-host spread. Systemic infection versus localized acute infections may be attributed to the virulence factor differences found within each of the clades. The complement control protein OPG032, the IL-1β interference genes within OPG201 and 208, and the cell-to-cell communication-interfering genes OPG015 and OPG195—all of which contribute to the increased case fatality rate in MPXV Clade I compared to Clade II. However, as we continue to see highly transmissible Clade I MPXVs arise, we should assume that increased virulence of Mpox is not tightly intertwined with a decrease in transmissibility (84). Instead, increased human-to-human transmissibility is likely attributed to codon optimization through human APOBEC editing through continual human-to-human transmission chains (69, 85). Smallpox, caused by the Variola virus, should warn us that MPXV may not remain a niche-transmitted pathogen—instead, through continued human adaptation, it may incite another global outbreak of Mpox.

While there is a large increase in human-to-human transmission, it is important to identify that there were multiple spillover events that began isolated transmission chains (86, 87). Therefore, novel strains of MPXV that were potentially more primed for human-to-human transmission were already circulating in small rodents prior to 2016. From an alternative viewpoint, there is the potential for the emergence of a well-adapted rodent MPXV to emerge or already have emerged. Enzootic spillover may have already occurred, but may not have been identified or sequenced yet (88). It is imperative to continue to survey, and predict, the perspective reservoir species to better anticipate the foundational genomic landscape of future MPXVs.

In conclusion, our review underscores the critical need to understand the genetic basis of MPXV adaptation and transmission dynamics. The ongoing epidemic in the Democratic Republic of the Congo, fueled by the Clade I MPXV lineage, highlights the urgency of this endeavor. By merging insights from Orthopoxvirus genetics and MPXV epidemiology, we have elucidated potential genetic drivers influencing host range, pathogenesis, and human-to-human transmission. Our analysis emphasizes the importance of MPXV genome reduction in shaping its virulence and transmission potential. Through comparative genomics and examination of host-range genes, key genetic determinants that distinguish MPXV from other Orthopoxviruses are noted, offering insight into its unique pathogenicity. Furthermore, our exploration of recent strains, such as Clade Ib and Clade IIb, sheds light on the rapidly evolving landscape of MPXV. Moving forward, elucidating the molecular mechanisms of the genes included in this review will be crucial for effective surveillance, prevention, and therapeutic intervention measures. By linking MPXV genotypes to specific phenotypes of public health importance, we can better predict and mitigate the risks posed by this emerging infectious disease. Our collective efforts in understanding MPXV genetics and epidemiology will be instrumental in safeguarding global health security against potential pandemic threats.

## Data Availability

All Genious files, including the sequences used in Fig. 2, can be found at the GitHub repository at https://github.com/BraydenYoung/MPXV_miniReview_mBio_BY. Requests for further information should be directed to the corresponding author.

## SUPPLEMENTAL MATERIAL

Fig. S1 msphere.00576-24-s0001.tiff Mauve genome alignment of Orthopoxviruses.

Legend msphere.00576-24-s0002.docx Legend to Fig. S1.

ASM does not own the copyrights to Supplemental Material that may be linked to, or accessed through, an article. The authors have granted ASM a non-exclusive, world-wide license to publish the Supplemental Material files. Please contact the corresponding author directly for reuse.
