# Supplementary figures and images for: Exploring the genomic basis of Mpox virus-host transmission and pathogenesis
Source: mSphere. 2024 Nov 14;9(12):e00576-24. doi: 10.1128/msphere.00576-24 (PMC11656787; doi:10.1128/msphere.00576-24)

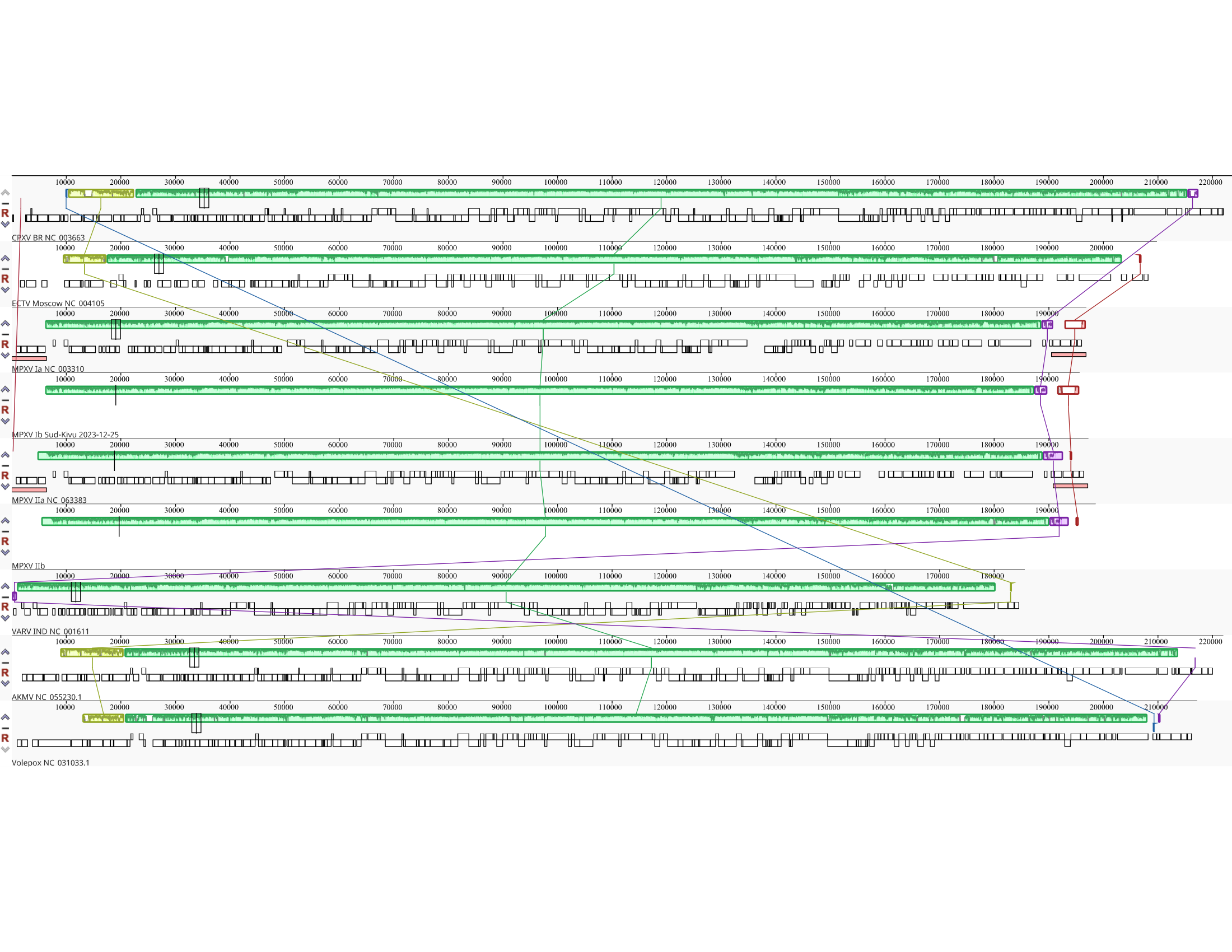

Supplement: Fig. S1 — Mauve genome alignment of Orthopoxviruses. [file msphere.00576-24-s0001.tiff]
