# Supplementary material for: Exploring the genomic basis of Mpox virus-host transmission and pathogenesis
Source: mSphere. 2024 Nov 14;9(12):e00576-24. doi: 10.1128/msphere.00576-24 (PMC11656787; doi:10.1128/msphere.00576-24)
Supplement: Legend — to Fig. S1. [file msphere.00576-24-s0002.docx]

**Figure S1. Mauve alignment of Orthopoxvirus genomes**

Orthopoxvirus genomes were genome aligned via progressiveMauve application within Genious. Genious files made available under Data Availability. Cowpoxvirus, CPXV, strain Brighton Red (NC_000363). Variola virus, VARV, (NC_001611) and Ectromelia virus, ECTV, (NC_004105). Clade Ia MPXV (NC_003310) and Clade IIa MPXV (NC_063383) from the Congo (DRC) and West Africa, respectively. MPXV Clade Ib, South Kivu, DRC(62). Clade IIb MPXV Rivers, 2022(73). Akhmeta virus AKMV (NC_055230.1) and Volepox virus VPXV (NC_031033.1).
